# A new world order: training clinicians for a new era in imaging

**DOI:** 10.2349/biij.2.4.e49

**Published:** 2006-10-01

**Authors:** RJ Hicks

**Affiliations:** Centre for Molecular Imaging, The Peter MacCallum Cancer Centre, Melbourne, Australia

There are three kinds of people – commonplace men, remarkable men, and lunatics (Mark Twain, *Following the Equator*).

Over the past 15 years or so I have had the distinction of being considered a member of each of the categories of humans identified by the great American humorist, author, and philosopher, Samuel Clemens (alias Mark Twain). My initial exposure to oncological PET as a fellow at the University of Michigan in the late 1980s and early 1990s and the seminal work of Professor Rich Wahl convinced me of the need to correlate PET images with more detailed anatomical studies. I was positive about the potential benefits of a combined device to achieve this end. Consequently, I suggested to an engineer of GE Medical Systems that his company should build such a device. His response was that they could not sell enough PET scanners to make money out of the business, and only a **lunatic** would suggest making an even more expensive device. In 1996, GE made an unsuccessful attempt to sell its PET business. In retrospect, I am sure that they would agree that this was one of the most fortuitous business strategy failures of all time. GE went on to release the first commercial PET/CT in 2001, followed soon after by Siemens. The latter had initially pioneered work in this field through the efforts of Professor David Townsend. Today, PET/CT is the most rapidly growing imaging modality in the world.

In 1996, coinciding with the nadir of corporate enthusiasm for PET, I established a PET facility at the Peter MacCallum Cancer Centre in Australia. This was done without Government capital or operational funding. The focus of our programme was to provide a clinical service to cancer patients. Many of my colleagues considered me a **lunatic**. PET, they told me, was a research tool, not a routine clinical imaging device. It was, they said, unlikely to be an affordable technique for cancer staging despite encouraging preliminary reports of its accuracy for this purpose. Despite this, by 1999, we had installed a second PET scanner. We were performing over 2000 clinical PET studies annually, and I was considered a **remarkable** man! As oncologists and imaging specialists became increasingly aware of the benefits of PET and more and more publications attested to its accuracy and patient management quality, it became evident that PET was a revolutionary approach to cancer evaluation. Modern oncology could not be practiced effectively without it. Again, I entered the ranks of the **commonplace** man since my vision had been self-evident.

When I first heard of the commercial development of hybrid PET/CT scanners, I was determined to gain access to this technology. My radiology colleagues, however, questioned the logic of adding an expensive CT to an already expensive PET scanner that could only do 10 to 20 scans per day. This appeared to them to be an incredible waste of a valuable resource when one could simply compare the PET and the CT images side by side. Nevertheless, a **lunatic** again, I persisted and in 2001 installed the first PET/CT in Australasia and one of the first in the world. It took little time for my colleagues to realise that this really was a **remarkable** advance. Today, after having used this technology for five years to perform over 10,000 scans, our clinicians can barely conceive of treating cancer patients without access to it. Indeed, PET/CT is now accepted by the clinical community throughout the world as an important, if not indispensable component of the diagnostic imaging armamentarium. My view of the imaging world has again reverted to the mean, and my perspectives are again **commonplace**.

It is now time, I feel, for me to revert to my **lunatic** self. I wish to consider the training of those who will be interpreting imaging studies in the future. I fear that I may make some statements that will evoke cries of protest. Nevertheless, I feel compelled to proceed. Who should be “credentialed” to read a new generation of hybrid imaging studies that will incorporate exquisite anatomical detail and previously unimagined molecular biological characterisation? Debate continues to rage around the world. Some time ago I attended a debate held at the Academy of Molecular Imaging in Madrid, Spain, where the nomenclature of hybrid imaging devices was discussed. Should it be a CT/PET as favoured by radiologists or PET/CT as supported strenuously by nuclear medicine physicians? At that time I was on a sabbatical and had performed microarrays to identify genes that were able to discriminate patients with oesophageal cancer who did and did not have a metabolic response to radiotherapy on PET. In the light of this experience, I facetiously made the suggestion that we should swab all the radiologists in the room and all the nuclear medicine physicians. From the collected DNA we would be able to see at which point in evolutionary history we had differentiated into such fundamentally different beings and the genes responsible. Perhaps, we could even find a gene product inhibitor to correct the deficiencies in one or other group (depending on your own particular bias). My point was that there is no fundamental difference among us. It is not nature, but nurture that creates different perspectives on what is important in imaging.

I recently read a wonderful book by North-American social anthropologist, Jane Jacobs. Titled “Dark Age Ahead,” this book provides a contrary view to the more widely held beliefs that the world is most threatened by ecological disaster due to global warming or global conflict ignited by religious beliefs. The author proposes what might seem a more prosaic and insidious forms of decay. Among the several factors she suggests as causing the gradual decline and the ultimate fall of western society, the one I found most resonant, was that our society has moved away from education towards credentialing. What exactly does that mean? She argues that education is open-ended, expansive, and unrestricted in its vision. It empowers the recipient to look beyond intellectual horizons, to imagine new ways of interacting with the world, and to build a system of thought and investigation that will expand possibilities into the future. Credentialing, on the other hand, is about defining what someone is capable of doing. It restricts endeavour and constrains perspectives. It limits opportunities for intellectual evolution and enquiry. Another important tenet of her book is that there has been a loss of self-regulation of the professions. She points out that ethical standards and maintenance of competence are no longer being an internalised character trait of professionals, but have to be regulated by external bodies. This is akin to having a police force to detect and punish crime on the assumption that it will occur. Accreditation Boards and credentialing bodies that are divorced from involvement in the field they purport to supervise run the risk of administering the law but not justice, conformity but not excellence.

How does this apply to imaging? In my view, PET/CT or CT/PET offers the medical community a great challenge; one that can degenerate into a battle for turf or one that can lead to a new era in imaging. In some parts of the world, this emerging modality is either controlled exclusively by radiologists or nuclear medicine physicians. In others, small subgroups within each of these specialty groups require further training to be credentialed. In Australia, for example, nuclear medicine physicians or radiologists with accreditation in both fields can apply for accreditation as a PET scan reader only if they spend a minimum of 20 working days in an approved training facility and review 300 cases under supervision. There is currently no provision for nuclear medicine physicians to gain accreditation to read the CT component, short of five years’ training in radiology. Therefore, the reader may ask, who should read PET/CT scans, radiologists or nuclear medicine physicians? My answer is neither and both! PET/CT is just one among a growing range of technologies that will blur the boundaries of structural and molecular imaging. We already have SPECT/CT, and prototype PET/MRI devices are being tested. A range of functional imaging techniques and new tracers will extend the range of information of MRI beyond anatomical detail. In addition, optical imaging is also entering the realms of clinical application.

I believe we need to focus on an entirely different skill set compared with the existing one in radiology or nuclear medicine, if we are to embrace and maximise the potential of these emerging modalities. Towards this end, I suggest we focus on a modular educational process that would enable practitioners to acquire the specific skill sets pertinent to their work environment. In the course of my work, I have noted the immense contribution of cardiologists towards nuclear cardiology, cardiac MRI and, more recently, multi-detector CT cardiac imaging. Neurologists have made a significant contribution towards neurologic SPECT and PET, and I believe that oncologists are doing the same in terms of oncologic PET/CT. I feel strongly that the success of our programme has been underpinned by my emphasis both on the need to learn about oncological principles and current treatment paradigms and to actively engage medical, surgical, and radiation oncologists in our programme to report oncological PET adequately. Consequently, I assert that we should rejoice in clinicians crossing specialty boundaries to bring new perspectives, unique skill sets, and differing knowledge bases to the practice of imaging. Does an oncologist or radiation oncologist wanting to use PET/CT for radiation treatment planning need to learn the nuances of obstetric ultrasound to do so? Should an epileptologist wanting to compare PET and MRI results with volumetric EEG be limited to getting opinions from imaging specialists who spend the vast majority of their day looking at chest X-rays? For that matter, do nuclear medicine physicians need to undergo a complete radiology training programme or radiologists a complete nuclear medicine training programme to read that component of a PET/CT that they are currently not credentialed to do? My answer is an unequivocal no! Rather, I suggest that we initiate training programmes that would enable development of specific modality expertise. All imaging specialists require a core skill set including knowledge of radiation safety and the physics of imaging. Apart from this, however, I believe there should be greater flexibility for clinicians to choose components from an increasingly complex array of techniques. We should encourage and recognise the efforts of trainees to gain clinical experience pertinent to their practice of imaging and imaging attachments for clinicians who will integrate this information into patient management. Many of my trainees over the years have come from clinical specialties, and I have encouraged others to also enter clinical training programmes. These trainees now practice a mix of clinical and imaging work and do both with greater insight and expertise than if they had constrained their training to one field.

Let us break down the hegemony of “learned colleges.” Let us not be ruled by bureaucrats. Let us develop a new way of training the imaging specialist of the future, to ensure a diversity and plurality of skills. *Vive la révolution*!

